# Local interleukin-10 production during respiratory syncytial virus bronchiolitis is associated with post-bronchiolitis wheeze

**DOI:** 10.1186/1465-9921-12-121

**Published:** 2011-09-12

**Authors:** Annemieke Schuurhof, Riny Janssen, Hanneke de Groot, Hennie M Hodemaekers, Arja de Klerk, Jan LL Kimpen, Louis Bont

**Affiliations:** 1 Laboratory for Health Protection Research, National Institute for Public Health and the Environment, Postbak 12 GBO, P.O.BOX 1, 3720 BA Bilthoven, The Netherlands; 2 Department of Pediatrics, Wilhelmina Children's Hospital, University Medical Center, Lundlaan 6, 3584 EA Utrecht, The Netherlands

**Keywords:** interleukin-10, lower respiratory tract infection, respiratory syncytial virus, wheeze

## Abstract

**Background:**

Respiratory syncytial virus (RSV) is the most common cause of bronchiolitis in infants. Following RSV bronchiolitis, 50% of children develop post-bronchiolitis wheeze (PBW). Animal studies have suggested that interleukin (IL)-10 plays a critical role in the pathogenesis of RSV bronchiolitis and subsequent airway hyperresponsiveness. Previously, we showed that ex vivo monocyte IL-10 production is a predictor of PBW. Additionally, heterozygosity of the single-nucleotide polymorphism (SNP) rs1800872 in the *IL10 *promoter region was associated with protection against RSV bronchiolitis.

**Methods:**

This study aimed to determine the *in vivo *role of IL-10 in RSV pathogenesis and recurrent wheeze in a new cohort of 235 infants hospitalized for RSV bronchiolitis. IL-10 levels in nasopharyngeal aspirates (NPAs) were measured at the time of hospitalization and the *IL10 *SNP rs1800872 genotype was determined. Follow-up data were available for 185 children (79%).

**Results:**

Local IL-10 levels during RSV infection turned out to be higher in infants that later developed physician diagnosed PBW as compared to infants without PBW in the first year after RSV infection (958 vs 692 pg/ml, p = 0.02). The *IL10 *promoter SNP rs1800872 was not associated with IL-10 concentration in NPAs.

**Conclusion:**

The relationship between high local IL-10 levels during the initial RSV infection and physician diagnosed PBW provides further evidence of the importance of the IL-10 response during RSV bronchiolitis.

## Background

Respiratory syncytial virus (RSV) is a negative-sense, single-stranded RNA virus and a member of the *Paramyxoviridae*, subfamily *Pneumovirinae*. RSV causes a wide range of clinical symptoms, varying from mild upper respiratory tract infection to severe bronchiolitis and pneumonia [1,2]. It is the most common cause of severe lower respiratory tract infection in children aged less than 1 year, and approximately 1-3% require hospitalization [3,4]. High-risk groups for severe RSV infection include infants with preterm birth, chronic lung disease of prematurity, congenital heart disease, cystic fibrosis, immunodeficiency disorders, and Down's syndrome [5,6]. Besides infants, specific adult populations are also at risk to develop severe RSV infection [7-10]. However, most infants hospitalized for RSV infection are previously healthy infants and do not fit the profile of a high risk patient [11]. RSV bronchiolitis is often followed by recurrent episodes of wheeze, in about 50% of cases, also referred to as post-bronchiolitis wheeze (PBW) [12-16]. PBW causes significant healthcare costs, and influences quality of life [17,18]. Since there is no effective therapy or vaccine for RSV infection, it is crucial for future management to characterize the precise mechanisms of this complex infection, including the development of PBW.

Identification of the functionality of genes that play an important role in disease susceptibility could enhance understanding of these mechanisms and disease development. The immunoregulatory effect of cytokine interleukin (IL)-10 on RSV infection has been widely investigated both in animal models [19,20] and human studies [21,22]. IL-10 is mainly produced by macrophages, monocytes, T cells, B cells, dendritic cells, mast cells and eosinophils. IL-10 can downregulate cytokine production by Th1-like T-cells and inhibit antigen presentation through downregulation of class II major histocompatibility complex antigens on monocytes [23,24]. Overexpression of IL-10 in the nasal mucosa of transgenic mice suppresses RSV replication in the respiratory tract [25], while ovalbumin-sensitized and challenged IL-10-/- mice develop airway hyperresponsiveness only if infected with RSV [26]. In RSV infected children IL-10 is elevated in nasopharyngeal secretions [27-29], while IL-10 is lower in atopic infants versus patients without atopy [30]. IL-10 plasma concentrations are higher in RSV infected hypoxic compared to non-hypoxic infants [31]. Previously, we have shown that IL-10 produced by monocytes of RSV infected infants is implicated in the occurrence of PBW [32]. Nonetheless, the relevance of IL-10 production in blood for the local response in RSV infected lungs is unknown. For the current study on the pathogenesis of PBW we have focused on the single-nucleotide polymorphism (SNP) rs1800872, which is located in the promoter region of the *IL10 *gene and therefore affects IL-10 production at the site of infection [33]. Heterozygosity of this SNP was associated with increased resistance to severe RSV infection [34]. Twin and family studies have shown that between 50% and 75% of the observed variability of IL-10 secretion is explained by genetic factors [35-37]. To clarify the role of IL-10 in the pathogenesis of PBW, the association between IL-10 levels during RSV infection and the *IL10 *promoter SNP rs1800872 with the development of PBW was investigated.

## Methods

### Study populations

The RSV-NPA study is a multicenter cohort study with previously healthy infants hospitalized with a first episode of RSV bronchiolitis before 13 months of age. Infants with Down syndrome, a history of wheezing, or cardiac or pulmonary pathology were excluded. Healthy infants with preterm birth were included. In total, 235 infants were included from October 2007 until March 2009 in fifteen large urban hospitals in The Netherlands (Flevo Hospital, Almere; Meander Medical Center, Amersfoort; Gelre Hospital, Apeldoorn; Alysis Rijnstate Hospital, Arnhem; Gelderse Vallei Hospital, Ede; Rivas Beatrix Hospital, Gorinchem; Jeroen Bosch Hospital, 's-Hertogenbosch; Tergooi Hospital, Blaricum; St. Antonius Hospital, Nieuwegein; Twee Steden Hospital, Tilburg; Diakonessen Hospital, Utrecht; Mesos Medical Center, Utrecht; University Medical Center, Utrecht; Maxima Medical Center, Veldhoven; Zuwe Hofpoort Hospital, Woerden). RSV infection was confirmed by positive immunofluorescence in epithelial cells from nasopharyngeal aspirates (NPAs) using the routine diagnostic procedures in the participating hospitals. Due to limited amount of NPA available for research, RSV detection was not confirmed by another method like PCR. All infants admitted to the pediatric intensive care unit (PICU) were intubated and mechanically ventilated, therefore stay at the PICU was identical to the requirement of mechanical ventilation. PBW was defined as a minimum of one wheezing episode diagnosed by a physician in the first year after hospitalization for RSV bronchiolitis. Follow-up data were available for 185 children (79%). If a general practitioner repeatedly did not respond to our questionnaire despite several attempts, PBW was classified as unknown. All parents of hospitalized infants agreed to participate and gave written informed consent. The study protocol was approved by the institutional review boards of all participating hospitals.

Controls (*n *= 1008) for genetic association were randomly taken from the Regenboog study, a large Dutch population health examination survey [38] as reported previously in the RSV-GENE study [39].

### Sample collection and marker detection

Sample collection and processing has been described previously [40] (and Schuurhof, et al, submitted). Undiluted NPAs of infants participating in the RSV-NPA study were aspirated within 24 hours after admission and stored at -80°C immediately. Subsequently, the NPAs were weighed, diluted, sonicated, and centrifuged. From 200 infants participating in the RSV-NPA study an adequate volume of NPA was available to measure the concentration of IL-10 using a commercial ELISA kit according to the manufacturer's instructions (Sanquin Reagents, Amsterdam, The Netherlands).

### DNA isolation and genotyping

In all infants participating in the RSV-NPA study buccal swabs were collected. DNA was isolated from buccal swabs using the QIAamp Blood Mini Kit (QIAgen, Venlo, The Netherlands). The DNA concentration was measured using the NanoDrop (Thermo Scientific, Breda, The Netherlands). SNP -592C/A, rs1800872 in *IL10 *was genotyped using a pre-designed Taqman SNP genotyping assay (C_1747363_10). For each sample 2.5 μl TaqMan Genotyping master mix, 0.25 μl TaqMan primer and 20 ng DNA were used in a total volume of 5 μl. The reaction was run on a 7500 Real Time PCR system (Applied Biosystems, Nieuwerkerk a/d IJssel, The Netherlands) according to the following protocol: 10 minutes incubation at 95°C, 40 cycles (15 s, 95°C; 1 min, 60°C), 4°C. Genotyping of the infants and controls participating in the RSV-GENE study was described previously [39]. Genotyping failed for 2 of the 235 infants in the RSV-NPA study. SNP rs1800872 was in Hardy-Weinberg equilibrium.

### Statistical analyses

Logarithmic transformation and logistic regression analysis were used to analyze the relationship between IL-10 levels and physician diagnosed PBW. Sample sizes needed to detect a significant difference were calculated. We used SD estimates from previous studies. With a = 0.05, b = 0.95, σ = 400 and IL-10 levels of 1000 and 750 in the 2 groups, a minimum of 67 children were needed in both groups. Polymorphism rs1800872 was analyzed for association with severe RSV disease, physician diagnosed PBW, and with the level of IL-10 in NPAs of RSV infected infants. Genetic analyses were performed with a χ^2 ^distribution of a 2 × 3 table (*df *= 2). Odds ratios determined on genotype level were based on comparing heterozygote infants (CA) versus major homozygote infants (CC). IL-10 levels among three genotypes were compared by using logarithmic transformation and one-way ANOVA testing. All analyses were performed using SPSS Statistics 18.0 (Chicago, USA). All hypothesis testing was two-sided, with a five percent threshold for statistical significance.

## Results

General characteristics of 235 RSV infected infants included in the RSV-NPA study are shown in Table 1. More boys than girls (55.7% and 44.3%, respectively) were included, the median age at hospitalization was two months, and 56.2% of the participating infants were diagnosed with PBW. We found no significant differences in baseline characteristics between infants with or without physician diagnosed PBW, or between our study population and infants for which follow-up data were lacking (*n *= 50).

**Table 1:** General characteristics of RSV infected infants with and without physician diagnosed PBW after RSV bronchiolitis

Characteristics	**Infants with PBW (*n *= 104)**^**1**^	**Infants without PBW (*n *= 81) **^**1**^	**Infants PBW unknown (*n *= 50) **^**1**^
Mechanically ventilated	12 (11.5%)	7 (8.6%)	3 (6.0%)
Mean length of hospitalization in days	6.1 (1-28)	5.2 (1-20)	5.8 (1-25)
Male gender	58 (55.8%)	41 (50.6%)	32 (64.0%)
Dutch nationality	74 (71.2%)	51 (63.0%)	32 (64.0%)
Median gestational age in weeks	39.9 (27.7-42.9)	39.6 (32.9-42.9)	39.0 (35.9-41.6)
Median age at infection in days	71 (8-375)	62 (10-372)	59 (10-308)
Mean parental atopy score^2^	1.8 (0-6)	1.7 (0-6)	2.1 (0-6)

All 235 infants included in the RSV-NPA study are shown in this table. Physician diagnosed post-bronchiolitis wheeze (PBW) in the first year after infection was not known in fifty infants and these infants were left out of PBW analyses. No statistical significant differences are present in the general characteristics of the infants with or without physician diagnosed PBW, or in the infants missing follow-up data (*n *= 50). ^1^Ranges and percentages are written between parentheses. ^2^A semiquantitative parental score for atopy was used [41]. One point was added to score for presence of each atopic symptom (eczema, hay fever, bronchitis, asthma, and food allergy) in both parents, with a minimum score of 0 and a maximum score of 10.

**Figure 1:**
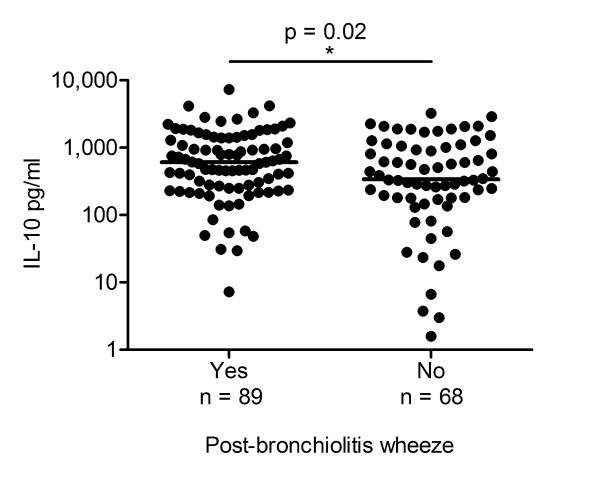
**IL-10 levels were higher in the NPAs of RSV infected infants with physician diagnosed PBW**. Levels of IL-10 were determined in nasopharyngeal aspirates (NPAs) of infants hospitalized for respiratory syncytial virus (RSV) bronchiolitis and analyzed after logarithmic transformation in groups with and without physician diagnosed post-bronchiolitis wheeze (PBW) in the first years after infection. NPAs were aspirated within 24 hours after admission to the hospital for RSV infection. Horizontal lines indicate median for analyzed groups. *Logistic regression analysis.

In our previous study, ex vivo monocyte IL-10 production during the convalescent phase of RSV bronchiolitis was shown to be higher in infants with PBW compared to infants without PBW [32]. To study whether this difference in IL-10 production is also seen in the local immune response, levels of IL-10 were analyzed in NPAs of RSV infected infants with and without physician diagnosed PBW in the first year after infection. NPAs were aspirated within 24 hours after admission to the hospital. IL-10 levels were higher in the NPAs of RSV infected infants who turned out to develop physician diagnosed PBW compared to infants without PBW in the year after RSV infection (958 vs 692 pg/ml, p = 0.02, see Figure 1).

**Figure 2:**
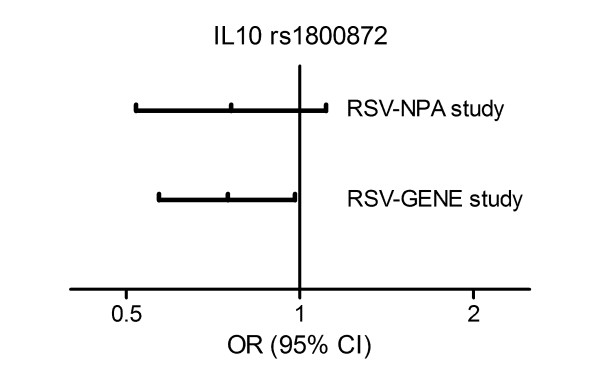
**Heterozygosity of *IL10 *promoter SNP rs1800872 is negatively associated with risk of severe RSV bronchiolitis**. Each line represents a different cohort, respectively the Dutch infants of the RSV-NPA study (*n *= 157), and the Dutch infants of the RSV-GENE study (*n *= 349) [39]. RSV infected infants in both cohorts were compared to the same group of population controls (*n *= 1008). Odds ratios (OR) and 95% confidence intervals (95% CI) were determined on genotype level, comparing heterozygote infants (CA) versus major homozygote infants (CC).

Previously, a significant association between SNP rs1800872 in the promoter region of the *IL10 *gene and severe RSV infection was found in the RSV-GENE study comparing 349 infants to 1008 population controls (p = 0.021, odds ratio (OR) 0.75 (95% confidence interval (CI) 0.57 - 0.98)), see Figure 2[39]. Heterozygosity was associated with reduced susceptibility to severe RSV infection. In the current study, a remarkably similar association was observed between this *IL10 *SNP and severe RSV infection comparing 157 Dutch infants of the RSV-NPA study with population controls (p = 0.056, OR 0.76 (95% CI 0.52 - 1.11)), see Figure 2. This association only reached borderline statistical significance, probably because this study was primarily powered to study the association between local IL-10 levels and physician diagnosed PBW.

**Figure 3:**
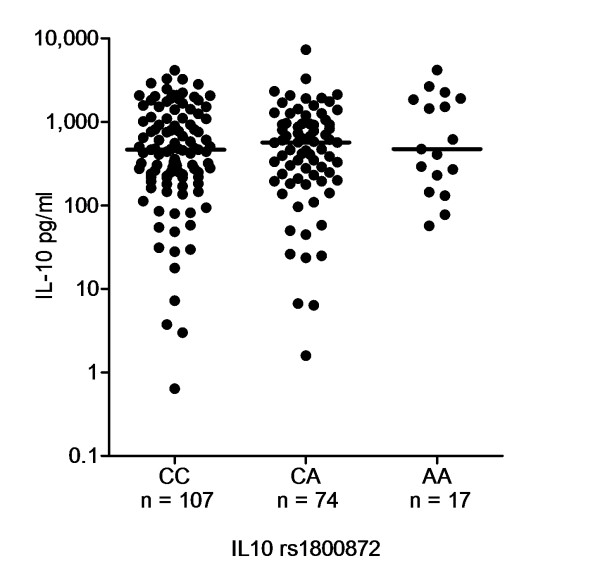
**IL-10 levels in NPAs of RSV infected infants did not differ based on *IL10 *genotype**. Levels of IL-10 were determined in undiluted nasopharyngeal aspirates (NPAs) of respiratory syncytial virus (RSV) infected infants and analyzed after logarithmic transformation. Horizontal lines indicate median for analyzed groups.

To study the relevance on the local immune response and the possible functional effect of this SNP in the promoter region of *IL10*, the levels of IL-10 were determined in NPAs of RSV infected infants. No difference was found in the levels of IL-10 among the three different *IL10 *genotypes (p = 0.67, see Figure 3). No association was observed between the *IL10 *SNP and physician diagnosed PBW, in children with the CC, AC, and AA genotype the incidence of PBW was 54.7%, 58.6%, and 56.3% (not significant), respectively.

## Discussion

The main finding of this study is that IL-10 levels in NPAs at the time of hospitalization for severe RSV infection were highest in infants who developed physician diagnosed PBW in the year after infection. These data indicate that the local immune response in infants at the time of acute, severe RSV infection differentiates infants with and without subsequent development of chronic airway morbidity. High IL-10 levels during severe RSV infection early in life affect airway morbidity later in life. If this relationship is causal, inhibition of IL-10 production during acute infection may alter the incidence of PBW. Further research is needed to study this relationship. Our current results are consistent with our previous finding that the production of IL-10 by monocytes after RSV infection is higher in patients with recurrent wheezing in follow-up than patients without wheezing [32]. Whether the different immune response at the time of RSV infection is the cause of physician diagnosed PBW or the consequence of a predefined susceptibility to infection is not yet known. Possibly, infants with higher IL-10 levels in NPA during acute RSV infection continue to have high levels of IL-10 during subsequent infections and develop more PBW.

Unlike previous publications [21,22,29,30], this is a prospective study which was performed in a large group of naturally RSV infected infants, all previously healthy and younger than 13 months of age. Levels of IL-10 at the time of acute RSV infection were combined with the development of PBW in infants in the year after RSV infection. Furthermore, the results of cytokine concentrations and genotyping in infants were compared instead of detecting mRNA in stimulated cell lines only [42-45].

Following candidate gene identification it is important to explore the functional consequences of the associated genetic variation [46]. SNPs located in promoter regions may change gene expression by altering transcription factor binding sites or by other more subtle mechanisms. Perrey et al. introduced putative high, intermediate, and low IL-10 producing haplotypes for three different *IL10 *promoter SNPs (rs1800896 (-1082G/A), rs1800871 (-819C/T), and rs1800872 (-592C/A) which is in complete linkage disequilibrium with rs1800871) [47]. Many other studies reported associations of these SNPs with altered transcriptional regulation of IL-10 for varying diseases [33,48-55]. Heterozygosity of the *IL10 *SNP rs1800872 was associated with increased resistance to severe RSV infection [34,39], suggesting that a balanced IL-10 response is required to reduce an excessive immune response, while allowing for a robust anti-viral immune response. However, no functional effect of this *IL10 *SNP on the local immune response could be detected, i.e. the levels of IL-10 in NPAs were comparable among the different genotypes of RSV infected infants. Whether there is truly no functional consequence of this SNP in the promoter region of *IL10 *during severe RSV infection is not known. We measured total IL-10 production in NPA irrespective of the source of IL-10, while there may be a cell type specific effect. Because protection against severe RSV infection was associated with heterozygosity of the *IL10 *SNP, it may be difficult to observe a functional effect of this promoter SNP. Alternatively, our cohort was too small to detect subtle differences. Especially the group homozygous for the minor allele consisted only of 17 infants. In literature, an advantage of the rs1800872 A allele has been associated to different infectious diseases, however, a specific heterozygous advantage of the CA genotype is not mentioned. Replication of this association has not been published to date. Both Wilson et al. and Helminen et al. reported no associations between eight SNPs in *IL10 *and RSV bronchiolitis [56,57]. However, in a subgroup analysis, two *IL10 *SNPs were associated with the need for mechanical ventilation [56]. In a small cohort, Gentile et al. showed that more RSV infected infants with a low IL-10 producing haplotype developed pneumonia compared to infants with an intermediate or high IL-10 producing haplotype [58]. Nevertheless, actual cytokine concentrations were not measured, and in another study with experimentally RSV challenged adults no correlation between haplotype and cytokine levels was observed [58,59]. As we analyzed only the SNP associated with severe RSV infection instead of three *IL10 *promoter SNPs needed to determine the haplotype, we could not compare our data to these studies [58,59]. Replication of findings from genetic association studies is required to exclude false positive associations. It has been difficult to replicate associations with severe RSV infection due to small sample size in studies, the related lack of power and the phenotypic heterogeneity between studies [60,61]. Therefore, more independent replication studies should be performed to confirm detected associations.

## Conclusion

Higher IL-10 levels in the airways of infants with RSV infection are observed in infants who subsequently develop physician diagnosed PBW, emphasizing the importance of regulation of the local immune response during RSV bronchiolitis.

## List of abbreviations used

CI: confidence interval; IL: interleukin; NPA: nasopharyngeal aspirate; OR: odds ratio; PBW: post-bronchiolitis wheeze; RSV: respiratory syncytial virus; SNP: single-nucleotide polymorphism.

## Competing interests

The authors do not have a commercial or other association that might pose a conflict of interest. No external financial support was provided for this study.

## Authors' contributions

AS included patients, collected samples, conceived and designed experiments, performed experiments, performed the statistical analysis, interpreted the data, wrote the manuscript. RJ conceived and designed experiments, interpreted the data, wrote the manuscript. HDG included patients, collected samples, performed experiments, analyzed the data. HH and ADK designed experiments, performed experiments, analyzed the data. JLLK conceived of the study, participated in its design, helped to draft the manuscript. LB conceived and designed experiments, interpreted the data, wrote the manuscript. All authors read and approved the final manuscript.
